# Medication errors associated with direct-acting oral anticoagulants: analysis of data from national pharmacovigilance and local incidents reporting databases

**DOI:** 10.1186/s40545-021-00369-w

**Published:** 2021-10-01

**Authors:** Abdulrhman Alrowily, Zahraa Jalal, Mohammed H. Abutaleb, Nermin A. Osman, Maha Alammari, Vibhu Paudyal

**Affiliations:** 1 School of Pharmacy, Institute of Clinical Sciences, College of Medical and Dental Sciences, Sir Robert Aitken Institute for Medical Research, University of Birmingham, Birmingham, B15 2TT UK; 2 Pharmaceutical Care Department, King Fahad Military Medical Complex (KFMMC), Medical Department, Ministry of Defence, Dhahran, Saudi Arabia; 3 Pharmaceutical Care Department, King Fahad Central Hospital, Jazan Health Affairs, Ministry of Health, Jazan, Saudi Arabia; 4 Department of Biomedical Informatics and Medical Statistics, Medical Research Institute, University of Alexandria, Alexandria, Egypt; 5 Pharmaceutical Care Department, King Abdulaziz Medical City, King Abdullah International Medical Research Center/King Saud, Bin Abdulaziz University for Health Sciences, Riyadh, Saudi Arabia

**Keywords:** Medication errors, Prescribing errors, Direct-acting anticoagulants (DOACs)

## Abstract

**Background:**

For more than a decade, direct oral anticoagulants (DOACs) have been approved in clinical practice for multiple indications such as stroke prevention in non-valvular atrial fibrillation treatment of deep vein thrombosis and pulmonary embolism. This study aimed to explore the nature and contributory factors related to medication errors associated with DOACs in hospital settings.

**Methods:**

Analysis of error reports using data from (a) Saudi Food and Drug Authority pharmacovigilance database and (b) local incidents reporting system from two tertiary care hospitals were included. Errors reported between January 2010 to December 2020 were also included. Statistical analyses were performed using IBM (SPSS) Statistics Version 24.0 software.

**Results:**

A total of 199 medication error incidents were included. The mean (range) age of affected patients was 63.5 (19–96) years. The mean reported duration of treatment when incidents happened was 90 days, with a very wide range from one day to 12 months. Prescribing error was the most common error type representing 81.4% of all errors. Apixaban was the most frequent drug associated with error reporting with 134 (67.3%) incidents, followed by rivaroxaban (18.6%) and dabigatran (14.1%). The majority of the patients (*n* = 188, 94.5%) showed comorbidities in addition to the conditions related to DOACs. Polypharmacy, an indication of treatment and duration of therapy were amongst the important contributory factors associated with errors.

**Conclusions:**

This observational study demonstrates the nature of DOAC related medication errors in clinical practice. Developing risk prevention and reduction strategies using the expertise of clinical pharmacists are imperative in promoting patient safety associated with DOAC use.

## Introduction

Direct oral anticoagulants (DOACs), or the non-vitamin K antagonist oral anticoagulants (NOACs) includes dabigatran, rivaroxaban, edoxaban, apixaban and betrixaban. In Saudi Arabia, clinicians follow recommendations of a recently published clinical pathway for DOACs by the Saudi Society of Clinical Pharmacy [1]. The Saudi Food and Drug Authority approved rivaroxaban in 2011, apixaban in 2014 edoxaban in 2018 and dabigatran in 2009 for stroke prevention in NVAF, and treatment or prevention of venous thromboembolism (VTE) [2]. Rivaroxaban also has other indications such as indefinite anticoagulation, stable coronary arterial disease or peripheral arterial disease, VTE prophylaxis in acutely ill medical patients and in total hip or knee arthroplasty. Apixaban and dabigatran also have approval for VTE prophylaxis in total hip or knee arthroplasty [1].

DOACs have gained popularity in clinical practice because they have fixed dosing and do not need frequent monitoring, have fewer drug and food interactions and offers less risk of bleeding compared to warfarin [3–9]. These characteristics have made them an attractive choice for the management of conditions requiring anticoagulation therapy [10]. They are labelled as high-risk medications due to the risk of causing significant patient harm if used inappropriately [11].

DOACs can cause bleeding if overdosed or exacerbate thromboembolic events if a therapeutic failure occurs, and it could further increase mortality [12]. Factors that could lead to inappropriate prescribing of DOACs could range from inaccurate dosing due to renal dysfunction, non-consideration of patients' body weight or age or concomitant polypharmacy [13]. Currently, there is a lack of research about medication errors associated with DOACs in Saudi Arabia [14]. Identifying DOACs most commonly involved with errors, type of errors, contributory factors, and patient characteristics that are linked with frequent occurrence of errors can enable identifying appropriate strategies to improve patient safety. This study aims to explore the types of medication errors and contributory factors associated with DOACs in hospital settings in Saudi Arabia using national and hospital local datasets. Specific objectives were to (a) identify the DOACs most commonly involved in the medication errors and (b) identify the stages in the medication journey where the errors occurred in order to understand the contributory factors.

## Methods

### Study design

The design of this study was a retrospective observational using incidents reporting databases from two healthcare institutions and the pharmacovigilance database of a national regulatory body, the Food and Drug Administration (FDA) in Saudi Arabia.

The hospital-based data were retrieved from the electronic medical record system for all errors reported from the year of such medications became available in Saudi Arabia, January 2010, to December 2020. They included patients’ demographic data (age, gender, weight, and height), drug name and its indication, comorbidities, dosing regimen and duration of treatment, type of medication error (prescribing, administration, and dispensing) and renal function test results.

The Pharmacovigilance Electronic Reporting Service in the Saudi FDA is an online spontaneous reporting system for reporting side effects, medication errors, and any defect in the quality of pharmaceutical preparations. It also allows sending and submitting communications to report a drug shortage by health practitioners, pharmacies, health institutions, companies, and community members. It receives voluntary reports of safety concerns from health practitioners and patients.

All reports associated with DOACs errors in adult population generated from 2010 to 2020 were included in the study for the final analysis. Reports without drug names or not related to DOACs were excluded. The local collaborators at the main centres in Saudi Arabia communicated with the respective departments to obtain the required medication error reports for the said period. The data were retrieved electronically from the institutions’ reporting systems using drug names as the search terms. The extracted data were cleaned for any duplications, and all variables were compiled in an excel file.

Statistical analyses were performed using IBM (SPSS) Statistics Version 24.0* software. Quantitative data (age in years) were described using median, range and Interquartile range after data distribution exploration normality using Kolmogorov–Smirnov (K–S test) and Shapiro tests. Descriptive analysis was undertaken to describe numbers and proportions. For all statistical tests, a significance level was determined below 5%. Monte Carlo exact test was used for comparing the categorical variables instead of the Chi-square test because the assumptions of conducting a Chi-square test were violated [> 20% of the cells had an expected count less than 5].

This study was reviewed and approved by a research ethics committee University of Birmingham (ERN_20-0551). Approvals were also obtained from King Abdullah International Medical Research Center (KAIMRC) at Ministry of National Guard, Saudi Arabia (SP/212/R), and from KFMMC (AFHER-IRB-2020-015), as well as from Saudi Food and Drug Authority (SFDA).

## Results

After reviewing the reported safety incidents of medication errors in all involved Saudi Arabia institutions, the database revealed that a total of 199 medication error incidents were found related to direct oral anticoagulants (DOAC); 109 errors were reported from the hospitals and 90 errors reported from Saudi Food and Drugs Authority (SFDA) from January 2010 to December 2020. Table 1 illustrates patients’ characteristics with the reported incidents.

**Table 1:** Characteristics of medical error reports included in the study

Sample characteristics (*n* = 199)	*n* (%)
Number of reported medical error/setting	
Hospitals	109 (54.8%)
SFDA	90 (45.2%)
Age in years: median (IQ range)	63.5 (48.0–76.0)
Gender: *n* = 198	
Male	99 (50.0%)
Female	99 (50.0%)
Weight in kg: median (IQ range)	75 (63.0–89.0)
Height in cm: median (IQ range)	161 (154.0–169.0)
Comorbidities	188 (94.5%)
DOAC type	
Apixaban	134 (67.3%)
Dabigatran	28 (14.1%)
Rivaroxaban	37 (18.6%)
Indications for DOAC	
Atrial fibrillation (AF)	113 (56.8%)
Deep vein thrombosis (DVT)	55 (27.6%)
Pulmonary embolism (PE)	20 (10.1%)
Cerebral venous thrombosis (CVT)	2 (1.0%)
Others	9 (4.5%)
DOAC dose at the incidence day: (only for the hospitals dataset)	
Not reported	2 (1.8%)
Under-dose	40 (36.7%)
Optimum dose	47 (43.2%)
Over-dose	6 (5.5%)
+ + Over-dose	14 (12.8%)
Medication error type	
Prescribing	162 (81.4%)
Dispensing	21 (10.6%)
Administration	9 (4.5%)
Monitoring	4 (2.0%)
Storage	3 (1.5%)
Medication error subtype	
Medication-duplicate therapeutic category	33 (16.6%)
Wrong patient	25 (12.6%)
Incorrect dose	44 (22.1%)
Incorrect frequency	29 (14.6%)
Incorrect quantity	30 (15.1%)
Incorrect duration	11 (5.5%)
Incorrect medication	9 (4.5%)
Delay preparation	4 (2.0%)
Improper/lack of documentation	4 (2.0%)
Side effects	2 (1.0%)
Contraindicated drug/drug interactions	8 (4.0%)
Duration of DOAC when incidence happened (days): median (IQ range)	90 (30–180)
e-GFR at the day of incidence (only for the hospitals)	
Mean ± SD	78.61 ± 33.21
Range	(0–173)

*SD* standard deviation, *IQ* interquartile range

### Medication errors related to specific DOAC and indication

**Fig. 1:**
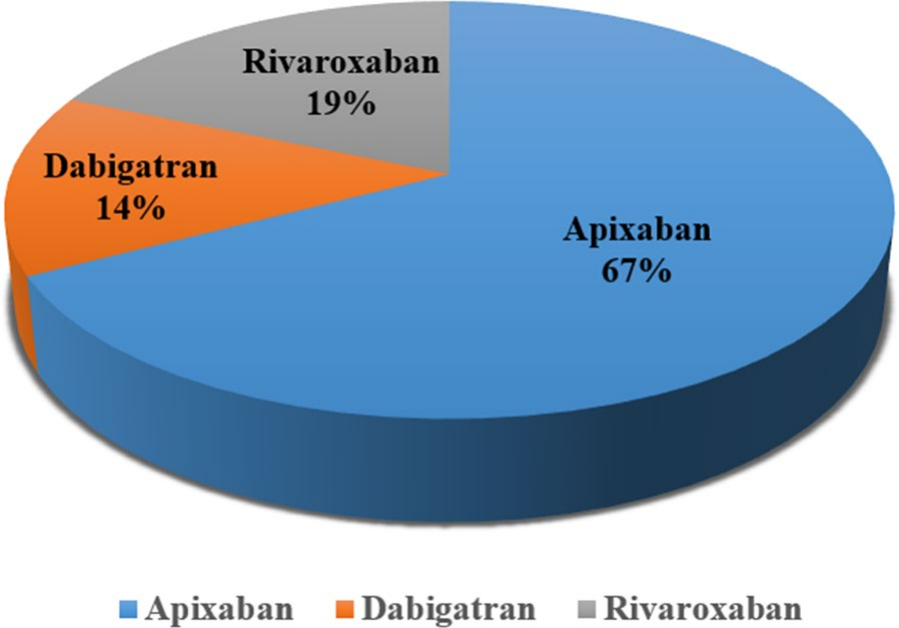
Proportions of direct-acting anticoagulants (DOACs) associated with medical errors

**Fig. 2:**
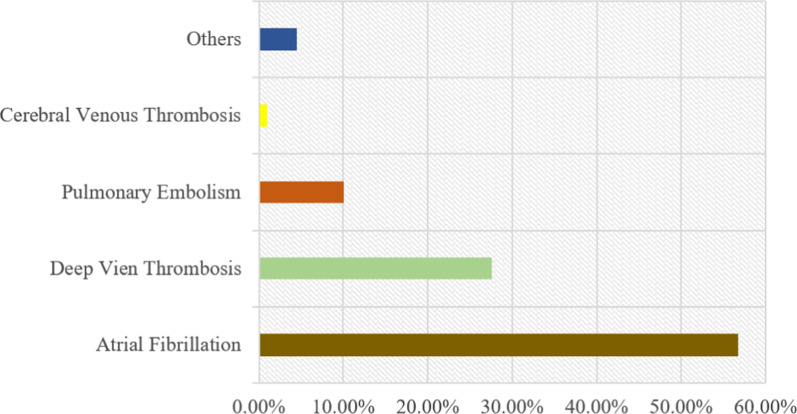
Different indications of direct oral anticoagulants (DOACs) in the medication error reports

Medication errors were reported for three DOAC drugs: the higher incidents were reported with apixaban (67%) followed by rivaroxaban (19%) and dabigatran (14%) as shown in Fig. 1. Most of the indications for DOACs were found to be related to atrial fibrillation at (56.8%), followed by deep vein thrombosis at (27.6%), pulmonary embolism at (10.1%), cerebral venous thrombosis at (1.0%), and others at (4.5%) as shown in Fig. 2.

### Errors as per the stages of medication journey

The most commonly identified subtype of prescribing errors related to prescribing incorrect dose accounting for 44 incidents (22.1%) followed by 33 (16.6%) incidents of duplicate prescribing, 30 (15.5%) incidents of dispensing incorrect quantity, prescribing incorrect frequency with (*n* = 29, 14.6%), 25 (12.6%) incidents of dispensing to the wrong patient and 11 (5.5%) incidents of prescribing an incorrect duration.

**Fig. 3:**
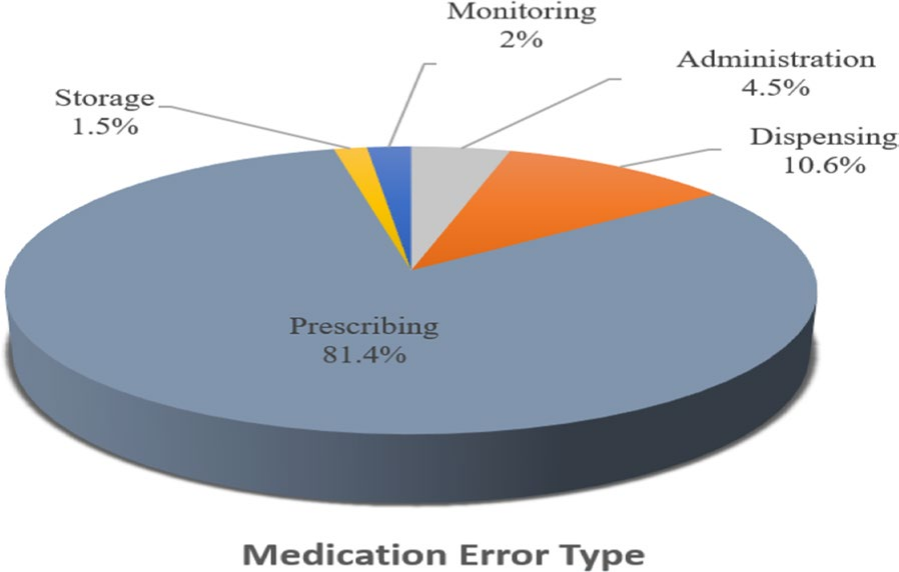
Different types of medication errors reported with direct oral anticoagulants (DOACs)

Figure 3 shows incidents of medication errors related to the stages of the medication use process.

**Fig. 4:**
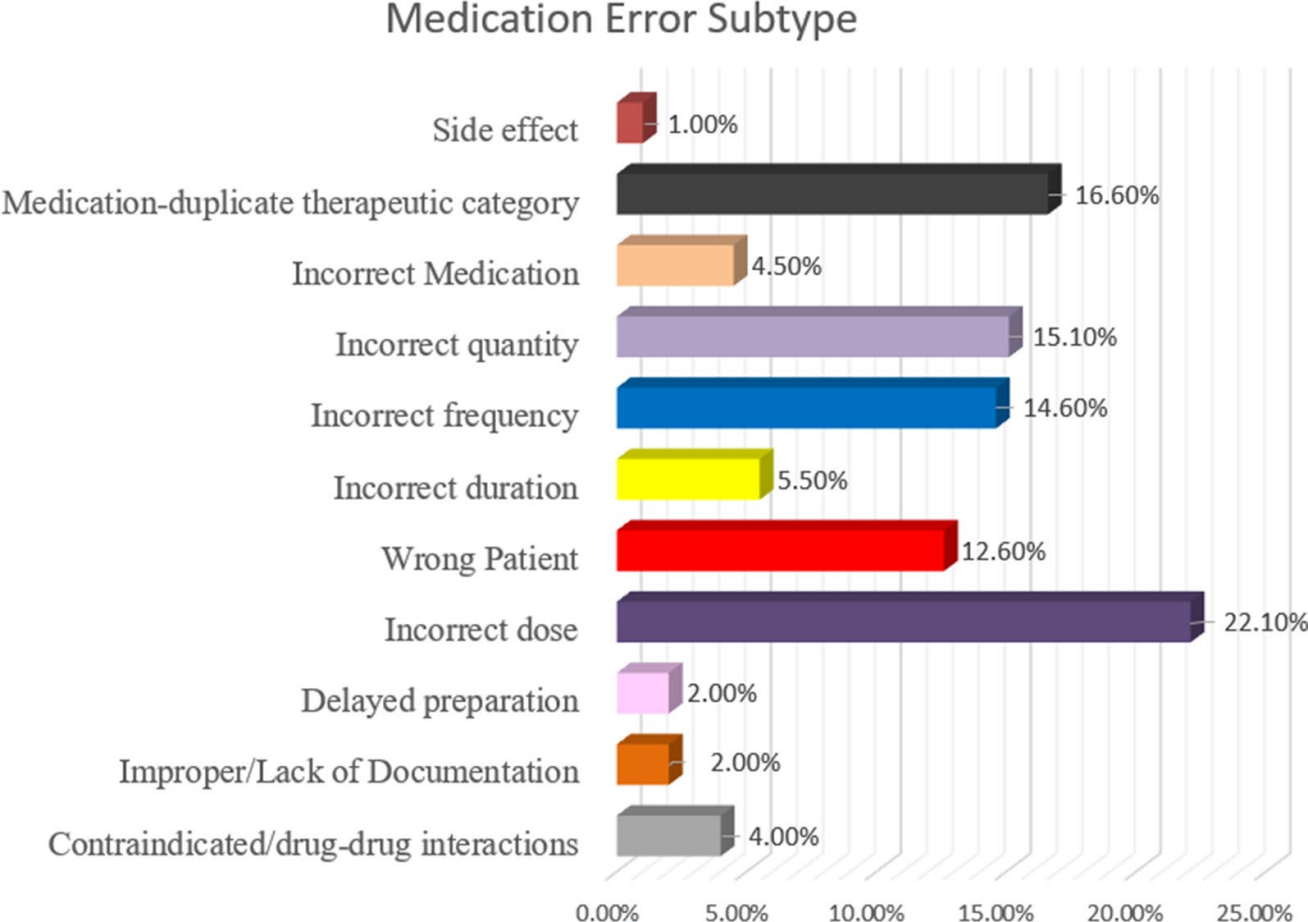
Sub-types of medication errors associated with direct oral anticoagulants (DOACs)

The other sub-types represented minimal incidents (less than 5%), such as delayed preparation, improper or lack of documentation. Figure 4 summarizes the percentages for all the identified types of medication errors.

### Errors classified as per specific DOAC and their indications

Whilst analysing the indication of the DOAC drugs among the collected reports, it was found that apixaban was mainly prescribed for atrial fibrillation (*n* = 73, 54.5%). It was also prescribed to the cases of deep vein thrombosis (*n* = 34, 25.4%), pulmonary embolism (*n* = 17, 12.7%), cerebral venous thrombosis (*n* = 2, 1.5%), and others such as hip or knee replacement surgery representing about 6%. Likewise, rivaroxaban was mainly prescribed for atrial fibrillation (*n* = 27, 73%), deep vein thrombosis (*n* = 8, 21.6%), and pulmonary embolism (*n* = 2, 5.4%). Dabigatran was prescribed equally for atrial fibrillation and deep vein thrombosis (*n* = 13, 46.4%, each), and also it was prescribed to single cases of pulmonary embolism and others, as shown in Table 2.

**Table 2:** Drug indication and the status of dose appropriateness of DOAC in the medical error reports

Drug indication			DOAC type			Statistical test*	*p*
			Apixaban (*n* = 134)	Dabigatran (*n* = 28)	Rivaroxaban (*n* = 37)		
		Atrial fibrillation	73 (54.5%)	13 (46.4%)	27 (73.0%)		
		Deep vein thrombosis	34 (25.4%)	13 (46.4%)	8 (21.6%)		
		Cerebral venous thrombosis	2 (1.5%)	0 (0.0%)	0 (0.0%)	12.883	0.115
		Pulmonary embolism	17 (12.7%)	1 (3.6%)	2 (5.4%)		
		Others	8 (6.0%)	1 (3.6%)	0 (0.0%)		

^*^Monte Carlo exact test: non-significant (*p* > 0.05)

### Errors classified as per specific DOAC and stages of medication journey

Whilst analysing the phases and types of medication errors associated with the three DOAC drugs, it was found that 103 (76.8%) of apixaban incidents occurred during the prescribing phase, 20 (15.0%) during the dispensing phase, 7 (5.2%) occurred during the administration phase, 3 (about 2.3%) occurred during the storage process, and only one incidents (about 0.7%) reported during the monitoring phase. For dabigatran, almost all the errors (*n* = 27, 96.7%) were within the prescribing phase, with only one reported incident during the dispensing phase. For rivaroxaban, the majority of errors (*n* = 32, 86.5%) occurred during the prescribing phase, with 3 (8.1%) incidents during the monitoring phase, and two incidents (5.4%) during the administration phase. There was a statistically significant difference in the percentage of medication errors among the three reported drugs during the prescribing phase (p = 0.001), while the other phases did not exert statistical significance, as shown in Table 3.

**Table 3:** Phases and types of reported medication errors associated with direct oral anticoagulants (DOAC) in the medical error reports

Medication error type	DOAC type			Statistical test	*p*
	Apixaban (*n* = 134)	Dabigatran (*n* = 28)	Rivaroxaban (*n* = 37)		
Prescribing (*n* = 162, 81.4%)					
Medication-duplicate therapeutic category	26 (19.4%)a	1 (3.6%)b	0 (0.0%)b		
Incorrect dose	17 (12.7%)a	5 (17.8%)a	11 (29.7%)a		
Wrong patient	9 (6.7%)a	3 (10.6%)a, b	8 (21.6%)b		
Incorrect frequency	23 (17.2%)a	2 (7.2%)a	3 (8.1%)a		
Incorrect quantity	11 (8.3%)a	9 (32.1%)b	9 (24.3%)b	46.262	0.001*
Contraindicated/drug–drug interactions	6 (4.4%)a	1 (3.6%)a	0 (0.0%)a		
Incorrect duration	5 (3.8%)a	4 (14.3%)a	1 (2.7%)a		
Incorrect medication	2 (1.4%)a	2 (7.2%)a	0 (0.0%)a		
Improper/lack of documentation	3 (2.2%)a	0 (0.0%)a	0 (0.0%)a		
Side effect	1 (0.7%)a	0 (0.0%)a	0 (0.0%)a		
Total per DOAC type	103 (76.8%)	27 (96.4%)	32 (86.5%)		
Dispensing (*n* = 21, 10.6%)					
Medication-duplicate therapeutic category	1 (0.7%)	0 (0.0%)	0 (0.0%)		
Incorrect dose	5 (3.8%)	0 (0.0%)	0 (0.0%)		
Wrong patient	5 (3.8%)	0 (0.0%)	0 (0.0%)		
Incorrect quantity	1 (0.7%)	0 (0.0%)	0 (0.0%)	21.000	0.140
Contraindicated/drug–drug interactions	0 (0.0%)	1 (3.6%)	0 (0.0%)		
Incorrect medication	5 (3.8%)	0 (0.0%)	0 (0.0%)		
Delay preparation	3 (2.2%)	0 (0.0%)	0 (0.0%)		
Total per DOAC type	20 (15.0%)	1 (3.6%)	0 (0.0%)		
Administration (*n* = 9, 4.5%)					
Medication-duplicate therapeutic category	5 (3.8%)	0 (0.0%)	0 (0.0%)		
Incorrect dose	1 (0.7%)	0 (0.0%)	1 (2.7%)		
Incorrect duration	0 (0.0%)	0 (0.0%)	1 (2.7%)	6.107	0.168
Side effect	1 (0.7%)	0 (0.0%)	0 (0.0%)		
Total per DOAC type	7 (5.2%)	0 (0.0%)	2 (5.4%)		
Monitoring					
Incorrect dosage form	1 (0.7%)	0 (0.0%)	2 (5.4%)		
Incorrect frequency	0 (0.0%)	0 (0.0%)	1 (2.7%)	4.00	0.504
Total per DOAC type	1 (0.7%)	0 (0.0%)	3 (8.1%)		
Storage (*n* = 3, 1.5%)					
Incorrect dose	1 (0.7%)	0 (0.0%)	0 (0.0%)		
Delayed preparation	1 (0.7%)	0 (0.0%)	0 (0.0%)	–	–
Improper/lack of documentation	1 (0.7%)	0 (0.0%)	0 (0.0%)		
Total per DOAC type	3 (2.3%)	0 (0.0%)	0 (0.0%)		

^*^Monte Carlo exact test: all phases were valid for conducting the analysis except for the storage phase because the dataset in the other phases was available for only one drug

Post hoc pairwise comparison using *z*-test was submitted: each subscript letter (a, b) denotes a subset of DOAC type categories whose column proportions differ significantly from each other on basis of medication error subtype with adjusted *p* value (< 0.167)

## Discussion

DOACs are considered high-risk medication and have the potential to cause harm when used erroneously [15]. This study explored the reported medication errors related to the three approved DOAC medications in the Kingdom of Saudi Arabia for the first time. Previous studies provide evidence of improper utilization associated with DOACs and potentially inappropriate prescribing leading to adverse events [15, 16]. In a retrospective study, medication errors were identified as a common root cause in 40% of anticoagulation-related adverse events [12].

In Saudi hospitals, several quality measures have been put in place at institutional levels, including restricting prescribing DOAC to certain specialities, e.g., (cardiology, haematology, internist, and through clinical pharmacy involvement). In addition, institutional guidelines have been developed based on different international guidelines, including American Heart Association and Chest guidelines. Furthermore, both the pharmacy department and the pharmacoeconomic centre monitor Medication Utilization Evaluation (MUE) of all DOAC listed in the hospital formulary. It is important that prescribers and healthcare professionals responsible for dispensing and administration of drugs are aware of the up-to-date guidelines and embrace additional safety practices when dealing with DOACs.

Among the three DOACs drugs, apixaban was the most frequently prescribed anticoagulant and hence associated with a higher number of errors. In our study, we were limited by the type of data that could be retrieved from the institutional safety reporting system, and this limited further investigation and follow-up for the seriousness of the reported medication errors. Previous studies have found that anticoagulants are often associated with underdosing and overdosing [17]. In one study, approximately a third of the patients received inappropriate dose including lower doses at (18%) and higher doses at (15%) [18].

This study also showed that DOACs-associated medication errors were most often reported during the prescribing phase. Most of these errors were typical and included wrong dose, medication, frequency, and duration. These results are in line with prior studies that found most reported medication errors occur in the prescribing stage. Raccah et al. found that prescribing errors with DOACs occur in nearly a third of the patients with AF [19]. Dreijer et al. observed that errors with anticoagulant therapy were most often seen during prescribing and administration phases [20]. In our study, the least type of errors reported were administration error, e.g., apixaban 5.2% and rivaroxaban (5.4%, which usually occurs during the administration of drugs by nurses in hospitals or by patients at home. In France, a prospective nationwide cohort study in primary care reported that 39% of the patients received at least one inappropriate DOAC prescription, mostly were inappropriate underdosing [21]. Another cross‐sectional study of oral anticoagulant use in patients with AF demonstrated underdosing in 13.1% of prescriptions, and unfortunately, 39.4% of their patients had not been prescribed an indicated DOACs [22].

This observational study is limited due to its retrospective design and the small number of variables available to undertake detailed evaluation of the nature and causes of errors. The sample size was limited despite including both national and local datasets suggesting the extent of under-reporting of medication errors. Furthermore, the consequences, including clinical, humanistic and cost-related outcomes of these errors, could not be estimated from the available data. Pharmacists and nurses are the main healthcare professionals reporting these prescribing errors in the institution, yet we do not have clear data on the reporting proportion.

## Conclusion

Further development and standardization of risk prevention measures is crucial to minimize the harm from medication errors with DOACs. Most of the reported DOACs-associated medication errors relate to prescribing stage, characteristically in the form of inappropriate dose, the inappropriate agent chosen, frequency, and duration. It is important to reinforce the education of healthcare professionals on the safe and effective use of DOACs, and the importance of medication error reporting. Pharmacists can play an integral role in minimizing medication error incidents through the development of safe and effective pharmacotherapeutic plans at the initial assessment and prescribing phase. Safety issues can also be identified and addressed during medicines reviews, medicines reconciliation and patient counselling. Healthcare professionals’ awareness and the need to have clear prescribing guidelines are imperative for safe DOACs prescribing.

## Data Availability

All data generated or analysed during this study are included in this published article.

## Abbreviations

UK: United Kingdom
DOACs: Direct oral anticoagulants; NOACs: Non-vitamin k antagonist oral anticoagulants
VTE: Venous thromboembolism
PE: Pulmonary embolism
DVT: Deep vein thrombosis
NVAF: Non-valvular atrial fibrillation
